# Long-Term Recovery Following Adolescent Anorexia Nervosa: A Mixed-Methods Follow-Up Study

**DOI:** 10.3390/nu18162606

**Published:** 2026-08-10

**Authors:** Izabela Łucka, Maciej Łucki, Patrycja Leśnicka, Marta Kopańska

**Affiliations:** 1 Department of Developmental Psychiatry, Psychotic Disorders and Old Age Psychiatry, Medical University of Gdansk, 80-210 Gdansk, Poland; izabelalucka@wp.pl; 2 School of Medicine, Collegium Medicum, University of Warmia and Mazury in Olsztyn, 10-719 Olsztyn, Poland; maciej.lucki@student.uwm.edu.pl; 3 Department of Medical Communication and Professional Competency Development, Faculty of Medicine, University of Rzeszów, 35-959 Rzeszów, Poland; patrycjazagata@gmail.com

**Keywords:** anorexia nervosa, nutritional rehabilitation, psychological recovery, orthorexia nervosa, psychiatric comorbidity, adolescence, long-term follow-up, treatment outcomes

## Abstract

**Background/Objectives:** Long-term recovery following adolescent anorexia nervosa (AN) remains incompletely understood because restoration of nutritional status does not necessarily coincide with psychological remission. Comprehensive evaluation of recovery requires consideration of psychological, behavioural, and patient-reported outcomes in addition to anthropometric measures. To evaluate long-term recovery following adolescent anorexia nervosa by integrating clinical outcomes, patient- and caregiver-reported experiences, orthorexic tendencies, and eating-disorder psychopathology, and to identify factors associated with persistent psychological impairment. **Methods:** This retrospective follow-up study employed a cross-sectional survey design. Families of 100 former patients previously hospitalized for adolescent anorexia nervosa in a child and adolescent psychiatric inpatient unit were invited to participate 5–10 years after discharge. Follow-up questionnaires were returned by 23 former patients and 17 caregivers. Former patients completed the SCOFF questionnaire and questions assessing orthorexic tendencies, whereas caregivers and the patients as well provided information regarding their child’s long-term recovery, treatment experiences, current functioning, and selected demographic characteristics. Responses from former patients and caregivers were analysed according to respondent group and the variables available for each group, while information from both groups was used to obtain a comprehensive assessment of long-term recovery. Descriptive and inferential statistical analyses were performed. **Results:** Mean BMI increased from 13.4 kg/m^2^ during the acute phase of illness to 18.8 kg/m^2^ at follow-up, indicating substantial nutritional improvement. Nevertheless, approximately 60% of former patients screened positive on the SCOFF questionnaire, suggesting persistent eating-disorder symptoms despite nutritional rehabilitation. Orthorexic tendencies were strongly associated with positive SCOFF screening results (Cramér’s V = 0.828; *p* < 0.001). Psychiatric comorbidity and poorer subjective health perception were also associated with less favourable long-term outcomes. Qualitative findings further demonstrated that recovery was perceived as extending beyond symptom reduction to include psychological wellbeing, restoration of autonomy, interpersonal functioning, and social reintegration.

## 1. Introduction

Defining recovery following anorexia nervosa (AN) remains one of the greatest unresolved challenges in eating disorder research. Despite substantial advances in diagnosis, nutritional rehabilitation, and multidisciplinary treatment, there is still no universally accepted definition of complete recovery. Traditionally, treatment success has been evaluated primarily using anthropometric indicators, particularly restoration of body weight and normalization of body mass index (BMI). Although these measures are essential for assessing medical stabilization and reversing the acute consequences of starvation, they do not adequately reflect the complexity of long-term recovery. Numerous studies have demonstrated that individuals who achieve a healthy body weight may continue to experience persistent eating disorder psychopathology, maladaptive eating behaviors, body image disturbances, impaired psychosocial functioning, and reduced health-related quality of life [1,2,3,4,5].

Anorexia nervosa is one of the most severe psychiatric disorders because of its complex biopsychosocial etiology, profound nutritional and metabolic consequences, and the highest mortality rate among mental illnesses. The effects of prolonged malnutrition extend far beyond weight loss, affecting endocrine, cardiovascular, gastrointestinal, skeletal, reproductive, and neurocognitive function, particularly when the disorder develops during adolescence—a critical period of physical growth, brain maturation, and psychosocial development. Consequently, recovery should no longer be regarded solely as the restoration of body weight but rather as a sustained process of biological, nutritional, psychological, and social adaptation. This evolving perspective has shifted contemporary research toward multidimensional models of recovery that extend beyond anthropometric outcomes and seek to better capture patients’ long-term health and functioning [6,7,8].

A major conceptual advance in eating disorder research was the recognition that recovery from anorexia nervosa cannot be adequately described using a single clinical or anthropometric indicator. This paradigm shift was largely driven by the multidimensional recovery model proposed by Bardone-Cone and colleagues, which defines complete recovery as the simultaneous restoration of physical, behavioral, and psychological health. Within this framework, physical recovery refers to normalization of nutritional status and body weight, behavioral recovery to the absence of restrictive eating, binge eating, purging, and other compensatory behaviors, whereas psychological recovery encompasses normalization of eating disorder-related cognitions, body image concerns, and dysfunctional attitudes toward food. Importantly, these domains do not necessarily recover in parallel. While restoration of body weight and eating behaviors may occur relatively early during treatment, cognitive rigidity, fear of weight gain, perfectionistic traits, and maladaptive beliefs about food often persist long after apparent clinical remission. Such residual symptoms are clinically relevant because they may impair quality of life, interfere with psychosocial functioning, and increase vulnerability to relapse despite satisfactory anthropometric outcomes. Consequently, recovery is increasingly conceptualized as a dynamic continuum rather than a dichotomous endpoint, requiring comprehensive assessment across biological, nutritional, behavioral, psychological, and social domains. This multidimensional perspective has fundamentally influenced contemporary clinical research and provides the conceptual foundation for evaluating long-term outcomes following anorexia nervosa [6,7,8,9,10,11]. The development of adolescent anorexia nervosa is considered to result from the interaction of multiple predisposing, precipitating, and perpetuating factors rather than from a single causal mechanism. Current evidence indicates that vulnerability arises through complex interactions between genetic susceptibility, neurobiological characteristics, psychological traits, family environment, and sociocultural influences. Genetic factors contribute substantially to disease risk, while psychological characteristics such as perfectionism, cognitive rigidity, obsessive-compulsive traits, anxiety, and intolerance of uncertainty frequently precede illness onset and may persist throughout recovery. Family-related factors, including high parental expectations, interpersonal difficulties, and exposure to stressful life events, may further increase vulnerability in susceptible individuals. During adolescence, these predispositions interact with profound neurodevelopmental, hormonal, and psychosocial changes, making this developmental period particularly sensitive to the emergence of restrictive eating behaviours. Sociocultural pressures emphasizing thinness, body dissatisfaction, social media exposure, and internalization of appearance ideals may additionally precipitate symptom onset in biologically vulnerable individuals. Contemporary models therefore conceptualize anorexia nervosa as a multifactorial disorder in which biological vulnerability interacts with developmental and environmental influences, contributing to substantial heterogeneity in both clinical presentation and long-term recovery trajectories [12,13,14,15].

From the perspective of nutritional sciences, recovery following anorexia nervosa extends considerably beyond the restoration of body weight and normalization of BMI. Nutritional rehabilitation is a complex and long-term process that aims not only to reverse the physiological consequences of prolonged starvation but also to restore adequate dietary intake, nutritional adequacy, metabolic homeostasis, and healthy eating behaviors that can be sustained over time. Successful rehabilitation requires normalization of energy availability, correction of macro- and micronutrient deficiencies, improvement in dietary diversity, and re-establishment of flexible eating patterns that support physical, cognitive, and psychosocial functioning. Consequently, nutritional recovery should be regarded as an essential and independent component of multidimensional recovery rather than merely a consequence of weight restoration.

Increasing evidence indicates that normalization of anthropometric parameters does not necessarily correspond to normalization of eating-related behaviors. Individuals who achieve satisfactory body weight frequently continue to avoid specific foods, rigidly adhere to self-imposed dietary rules, excessively monitor food composition, or experience marked anxiety when their eating patterns deviate from personally established standards. Although these behaviors may initially appear compatible with a healthy lifestyle, persistent dietary rigidity and excessive cognitive preoccupation with food can compromise nutritional adequacy, reduce dietary flexibility, and adversely affect long-term quality of life. Therefore, contemporary follow-up studies increasingly recommend complementing conventional anthropometric outcomes with assessments of dietary quality, eating behaviors, food-related cognitions, and nutritional functioning to obtain a more comprehensive evaluation of long-term recovery. Such an approach is particularly relevant in adolescents and young adults, in whom adequate nutrition plays a fundamental role in supporting continued physical development, endocrine recovery, bone health, cognitive performance, and psychosocial adaptation [1,3,12,13].

Within this multidimensional framework, increasing attention has been directed toward persistent eating-related cognitions and behaviors that may remain evident despite apparent clinical remission. One phenomenon attracting growing scientific interest is the presence of orthorexic tendencies, defined as an excessive preoccupation with consuming foods perceived as healthy, accompanied by rigid dietary rules, compulsive food selection, and considerable emotional distress when these self-imposed standards cannot be maintained. Although orthorexia nervosa has not yet been formally recognized as a distinct psychiatric disorder, accumulating evidence indicates substantial overlap with established eating disorders, particularly anorexia nervosa. Shared characteristics include perfectionism, cognitive rigidity, obsessive-compulsive traits, excessive dietary restraint, and an overvaluation of food-related behaviors, suggesting that orthorexic tendencies may represent one manifestation of persistent eating disorder psychopathology rather than an entirely separate clinical entity [14,15,16,17].

This perspective is especially relevant in individuals recovering from anorexia nervosa. Behaviors that initially appear to reflect successful nutritional rehabilitation—such as careful food selection or increased attention to diet quality—may gradually become excessively restrictive and inflexible, resulting in persistent anxiety surrounding food, avoidance of entire food groups, and impaired social functioning. Consequently, apparent physical recovery may coexist with clinically meaningful disturbances in eating-related cognitions and behaviors that remain undetected when recovery is assessed solely on the basis of body weight or BMI. Evaluating orthorexic tendencies may therefore provide complementary information regarding the quality and completeness of recovery, particularly when interpreted alongside nutritional, psychological, and behavioral outcomes rather than as an isolated diagnostic construct. This integrative perspective is consistent with the growing view that sustainable recovery requires not only nutritional adequacy and medical stability but also cognitive flexibility, adaptive eating behaviors, and the ability to maintain a balanced relationship with food over time.

Despite substantial progress in understanding the long-term course of anorexia nervosa, important gaps remain in the assessment of recovery. Most longitudinal studies have focused on isolated clinical outcomes, including weight restoration, relapse rates, or remission of core eating disorder symptoms, whereas relatively few have adopted an integrated framework encompassing nutritional, psychological, behavioral, and psychosocial dimensions simultaneously. Furthermore, recovery has most often been evaluated from either the clinician’s or the patient’s perspective, while the experiences and observations of caregivers have received considerably less attention. This represents an important limitation, particularly in individuals treated during adolescence, where family involvement constitutes a fundamental component of both treatment and long-term rehabilitation.

Caregivers frequently observe everyday eating behaviors, dietary flexibility, emotional functioning, and social adaptation that may not be fully captured by anthropometric measures or patient self-reports alone. Conversely, former patients provide unique insight into their subjective experiences, cognitive recovery, and quality of life after treatment. Integrating these complementary perspectives may therefore provide a more comprehensive understanding of long-term recovery, identify subtle residual difficulties that remain clinically relevant despite apparent remission, and improve the interpretation of post-treatment outcomes. Such an approach is consistent with the multidimensional recovery model and reflects the growing emphasis on patient-centered and family-oriented care in eating disorder research.

Against this background, the present study was undertaken to comprehensively evaluate long-term multidimensional recovery among individuals previously hospitalized for adolescent anorexia nervosa. Rather than defining recovery solely by anthropometric improvement, we adopted an integrated framework encompassing physical, nutritional, behavioral, psychological, and psychosocial dimensions of post-treatment functioning. Particular emphasis was placed on nutritional recovery and orthorexic tendencies as potential indicators of persistent eating-related psychopathology, as well as on complementary evaluations provided by former patients and their caregivers. By combining objective and subjective measures of long-term outcome, this study seeks to provide a more comprehensive understanding of recovery following adolescent anorexia nervosa and to contribute to the development of clinically meaningful follow-up strategies that extend beyond weight restoration alone.

## 2. Materials and Methods

### 2.1. Study Design

This retrospective follow-up study was conducted to evaluate long-term multidimensional recovery among individuals previously hospitalized for adolescent anorexia nervosa (AN). The study protocol was approved by the appropriate Ethics Committee in 2015 (NKBBN/278/2014 on 4 May 2015), prior to participant recruitment, and the study was conducted in accordance with the ethical principles of the Declaration of Helsinki [18]. A cross-sectional follow-up survey was conducted 5–10 years after discharge from inpatient psychiatric treatment to assess long-term physical, psychological, behavioral, and psychosocial outcomes. The study adopted a mixed-methods approach, integrating objective clinical indicators with patient- and caregiver-reported outcomes. This design was selected to provide a comprehensive assessment of recovery, acknowledging that nutritional rehabilitation and body weight restoration alone do not fully reflect psychological wellbeing, social functioning, or subjective perceptions of recovery. Former patients treated in the Child and Adolescent Psychiatric Inpatient Unit and, whenever possible, one parent or primary caregiver were invited to participate in the follow-up survey. Including both perspectives enabled a broader evaluation of long-term recovery and post-treatment functioning.

### 2.2. Participants

The study population consisted of 100 former patients who had been hospitalized for anorexia nervosa during adolescence in the Child and Adolescent Psychiatric Inpatient Unit of the Medical University of Gdańsk. Eligible participants were individuals with a diagnosis of anorexia nervosa who had completed inpatient treatment and had been discharged 5–10 years before the follow-up survey. Individuals with eating disorders other than anorexia nervosa and those whose follow-up period was shorter than 5 years or longer than 10 years were not eligible for inclusion.

A follow-up questionnaire was mailed to all eligible former patients identified from the hospital database. Each invitation also included a separate questionnaire for one parent or primary caregiver. Participation was voluntary, and completed questionnaires were returned by mail.

Completed questionnaires were received from 23 former patients (aged 20–32 years) and 17 parents or primary caregivers (aged 47–68 years), yielding an overall response rate of 40%. In addition, reliable information was obtained regarding three former patients who had died due to severe somatic complications of anorexia nervosa; these cases were reported descriptively but were not included in the statistical analyses.

Some returned questionnaires contained incomplete responses. However, all questionnaires containing analyzable data were retained. Consequently, the number of observations differs across individual analyses according to the availability of responses for specific variables. Responses from former patients and caregivers were analysed according to respondent group and the variables available for each group, while information from both groups was used as complementary sources to provide a comprehensive assessment of long-term recovery.

### 2.3. Data Collection

Long-term outcomes were assessed using a structured follow-up questionnaire developed specifically for the present study. The instrument was designed to evaluate multidimensional recovery and consisted of four domains. The first domain collected demographic and clinical information. Former patients provided information regarding current age, age at illness onset, duration of untreated illness before first hospitalization, current body weight and height (used to calculate BMI), psychiatric comorbidity, and subjective health status. Caregivers completed selected demographic questions concerning themselves and provided information regarding their child’s current health and long-term functioning. The second domain evaluated experiences of inpatient treatment as reported by former patients and caregivers. Respondents indicated which therapeutic interventions had been received during hospitalization, including nutritional rehabilitation, supervised meals, body weight monitoring, pharmacotherapy, individual psychotherapy, group psychotherapy, family therapy, psychoeducation, therapeutic community activities, and therapeutic contracts. Participants subsequently rated the perceived effectiveness of each intervention using a five-point Likert scale (1 = least effective; 5 = most effective) and provided an overall evaluation of hospitalization. The third domain was completed exclusively by former patients and included the SCOFF questionnaire, a validated five-item screening instrument developed by Morgan et al. Consistent with established recommendations, two or more affirmative responses indicated a positive screening result suggestive of clinically significant eating-disorder symptoms requiring further clinical assessment. The fourth domain, completed by former patients, explored orthorexic tendencies, including excessive concern with healthy eating, food quality, and rigid dietary practices. Because the primary objective was to evaluate clinically relevant long-term eating-related behaviours rather than diagnose orthorexia nervosa, a limited number of targeted questions based on Bratman’s conceptual framework was included instead of a validated orthorexia questionnaire. This approach minimized respondent burden after a long follow-up period while allowing assessment of clinically relevant orthorexic tendencies.

### 2.4. Outcome Measures

The primary outcome was long-term multidimensional recovery, evaluated using integrated anthropometric, nutritional, psychological, behavioral, and psychosocial indicators. Anthropometric recovery was assessed using current BMI calculated from self-reported body weight and height obtained at follow-up. Psychological and behavioral functioning was assessed using the SCOFF questionnaire as a screening instrument for persistent eating-disorder symptoms, whereas psychosocial recovery was evaluated through participants’ subjective assessment of health, recovery, and current functioning. Secondary outcomes included perceived effectiveness of inpatient therapeutic interventions, long-term nutritional status, psychiatric comorbidity, overall evaluation of hospitalization, and subjective treatment effectiveness. Exploratory analyses examined orthorexic tendencies as potential markers of persistent eating-disorder psychopathology. Associations between orthorexic tendencies and BMI, SCOFF results, psychiatric comorbidity, and subjective recovery were investigated to determine whether rigid healthy-eating behaviors persisted despite apparent clinical recovery.

### 2.5. Statistical Analysis

Continuous variables are presented as means and standard deviations (SD) or medians and interquartile ranges (IQR), depending on data distribution, whereas categorical variables are presented as frequencies and percentages. The normality of continuous variables was assessed using the Shapiro–Wilk test. Group comparisons were performed using Student’s *t*-test or the Mann–Whitney U test, as appropriate. Associations between categorical variables were evaluated using the χ^2^ test or Fisher’s exact test. The strength of associations identified in contingency tables was quantified using Cramér’s V coefficient, interpreted as follows: <0.10 negligible, 0.10–0.29 weak, 0.30–0.49 moderate, 0.50–0.69 strong, and ≥0.70 very strong. Ratings of therapeutic interventions were analyzed descriptively. Because these analyses were exploratory, results are presented alongside other clinical and psychosocial outcome measures. Because some returned questionnaires contained incomplete responses, analyses were performed using all available data without imputing missing values. Consequently, the number of observations varied across individual analyses depending on the availability of responses for individual variables. All statistical analyses were two-tailed, and a *p*-value < 0.05 was considered statistically significant. Statistical analyses were performed using IBM SPSS Statistics (Version 29; IBM Corp., Armonk, NY, USA) [19].

## 3. Results

### 3.1. Clinical Characteristics and Long-Term Outcomes

The follow-up survey was completed by 23 former patients previously hospitalized for adolescent anorexia nervosa (AN) and 17 parents or primary caregivers. Data from former patients and caregivers were analysed according to respondent group and the variables available for each group, while information from both groups was used to provide complementary perspectives on long-term recovery. The mean age at illness onset was 14.5 years, while the average interval between symptom onset and the first psychiatric hospitalization was 1.2 years. During the acute phase of illness, the mean lowest body mass index (BMI) was 13.4 kg/m^2^, indicating severe malnutrition. At follow-up, the mean BMI increased to 18.8 kg/m^2^, reflecting substantial nutritional improvement (Table 1).

Despite this improvement, restoration of body weight did not consistently correspond to complete clinical recovery. More than half of the respondents (54%) remained underweight at follow-up, and approximately 60% of former patients screened positive on the SCOFF questionnaire, suggesting the persistence of eating-disorder symptoms despite partial normalization of BMI. These findings suggest that anthropometric recovery alone does not adequately reflect long-term recovery from anorexia nervosa and support the need for multidimensional outcome assessment.

Follow-up information additionally identified three deaths attributable to severe complications of anorexia nervosa, including one due to sepsis secondary to urinary tract infection and two resulting from circulatory failure. Although these individuals were not included in the statistical analyses, these observations emphasize the potentially fatal long-term consequences of AN and highlight the importance of prolonged multidisciplinary follow-up after inpatient treatment.

### 3.2. Prognostic Factors Associated with Recovery and Positive SCOFF Screening Results

To identify factors associated with long-term recovery, relationships between selected clinical and behavioral variables and positive SCOFF screening results were examined among former patients. The overall pattern of prognostic factors is summarized in Table 2, whereas detailed associations are presented in Table 3, Table 4 and Table 5.

The strongest relationship was observed between orthorexic tendencies and positive SCOFF screening results among former patients (Table 3). Former patients reporting persistent preoccupation with healthy eating and dietary purity were significantly more likely to screen positive for positive SCOFF screening results than those without such concerns (Fisher’s exact test, *p* < 0.001; Cramer’s V > 0.800). This finding suggests that orthorexic tendencies may represent an important marker of persistent eating-disorder symptoms identified by the SCOFF screening questionnaire during long-term recovery.

Psychiatric comorbidity also showed a strong association with unfavorable long-term outcomes (Table 4). Respondents with coexisting psychiatric disorders, particularly depressive disorders, were substantially more likely to report persistent orthorexic behaviors than participants without additional psychiatric diagnoses (Cramer’s V = 0.690), suggesting that psychiatric comorbidity contributes to the persistence of maladaptive eating-related behaviors after treatment.

Self-rated health demonstrated a significant protective relationship with recovery (Table 5). Former patients who perceived themselves as fully recovered were significantly less likely to obtain positive SCOFF screening results than those reporting incomplete recovery (Fisher’s exact test, *p* = 0.006; Cramer’s V = 0.626), indicating that subjective health perception represents an important complementary indicator of long-term recovery.

Among former patients, lower BMI during the acute phase of illness remained positively correlated with lower BMI at follow-up (Pearson’s r = 0.602), indicating that greater severity of malnutrition at illness onset continued to influence nutritional status several years after treatment.

Taken together, these findings demonstrate that long-term recovery following adolescent anorexia nervosa extends beyond restoration of body weight and is closely associated with persistent eating-disorder symptoms identified by the SCOFF screening questionnaire, psychiatric comorbidity, and subjective health perception.

### 3.3. Therapeutic Interventions Applied During Hospitalization

Former patients and caregivers consistently reported that inpatient treatment had been based on a multidisciplinary therapeutic model integrating nutritional rehabilitation, medical supervision, pharmacotherapy, and psychotherapeutic interventions.

Among former patients, supervised meals and regular body weight monitoring were the most frequently reported treatment components, followed by prescribed dietary management, therapeutic community activities, group psychotherapy, therapeutic contracts, and individual psychotherapy. Less frequently reported interventions included psychoeducation, social skills training, psychomotor activities, and educational support.

Caregivers described a comparable treatment profile. Supervised meals, prescribed dietary management, therapeutic contracts, and body weight monitoring were perceived as central elements of inpatient care. Family therapy and temporary separation from family members were also frequently reported, whereas psychoeducation and complementary therapeutic activities were less commonly indicated.

**Figure 1:**
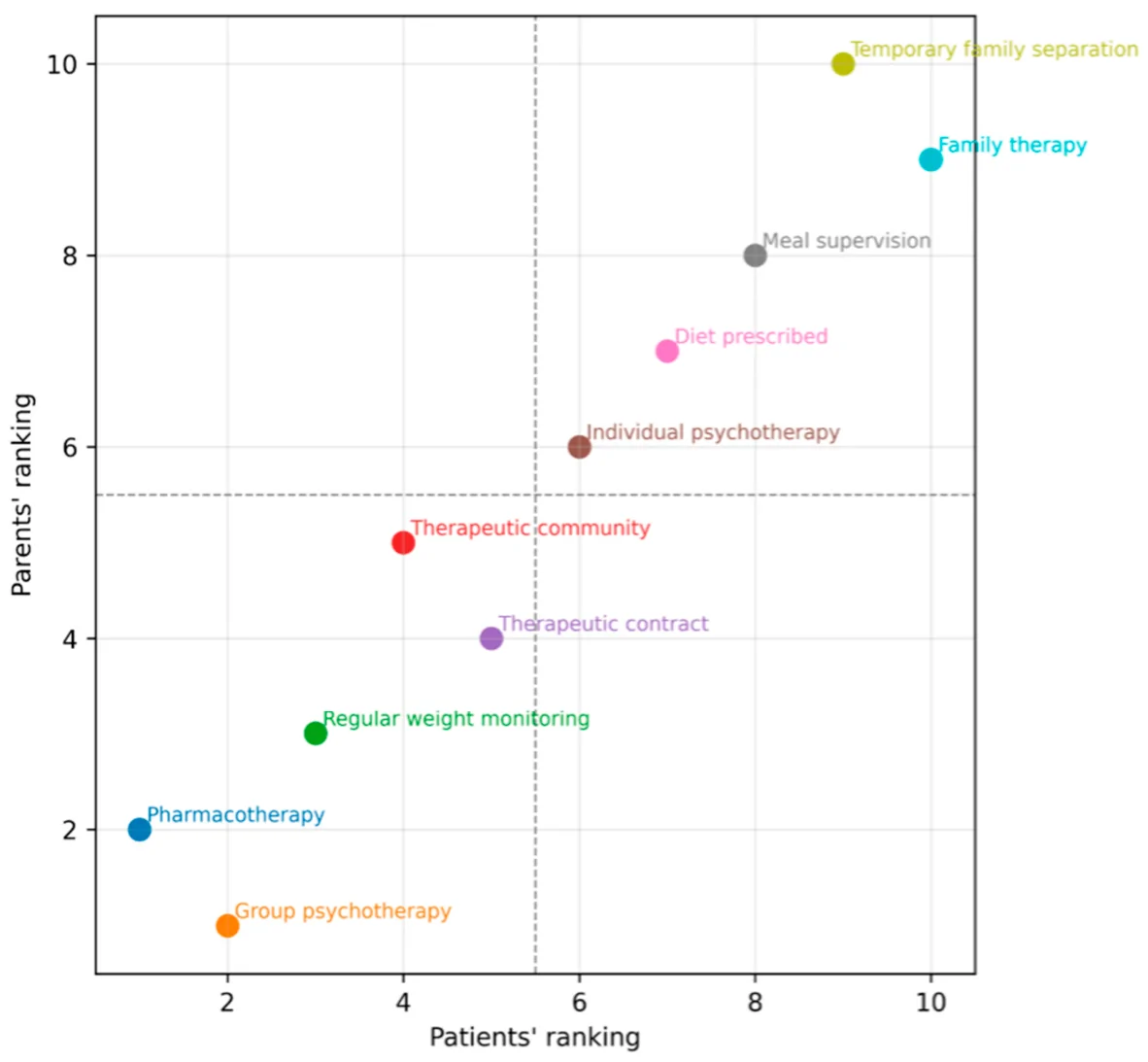
Agreement Between Former Patients and Caregivers in Ranking the Relative Importance of Inpatient Therapeutic Interventions.

To evaluate the consistency of respondents’ recollections, rankings of therapeutic interventions reported by former patients and caregivers were compared (Figure 1). Spearman’s rank correlation demonstrated a strong positive association between both groups (ρ = 0.858, *p* < 0.001), indicating substantial agreement regarding the relative importance of therapeutic procedures implemented during hospitalization.

Although caregivers tended to assign greater importance to behavioral supervision, including body weight monitoring and temporary separation from family members, former patients more frequently emphasized psychotherapeutic components of treatment. Nevertheless, the overall ranking of interventions remained highly consistent across both respondent groups, supporting the consistency of treatment-related evaluations reported independently by former patients and caregivers.

### 3.4. Perceived Effectiveness of Therapeutic Interventions

Former patients and caregivers evaluated the perceived effectiveness of individual therapeutic interventions received during hospitalization using a five-point Likert scale. The integrated ratings obtained from former patients and caregivers are presented in Table 6.

Overall, pharmacotherapy received the highest mean effectiveness score (3.97), closely followed by group psychotherapy (3.96) and regular body weight monitoring (3.91). These interventions were consistently regarded as the most beneficial components of inpatient treatment.

Moderately high effectiveness ratings were assigned to therapeutic community activities (3.75), therapeutic contracts (3.75), individual psychotherapy (3.64), dietary management prescribed by healthcare professionals (3.60), and meal supervision (3.51). These findings suggest that respondents perceived recovery as the result of a coordinated multidisciplinary treatment approach rather than the effect of a single therapeutic modality.

The lowest ratings were assigned to temporary separation from family members (3.19) and family therapy (3.16). Nevertheless, both interventions remained above the midpoint of the rating scale, indicating that participants generally perceived all components of inpatient treatment as contributing positively to recovery.

Although some differences were observed between former patients and caregivers regarding the relative importance of specific interventions, both groups consistently identified comprehensive multidisciplinary care as the cornerstone of successful treatment.

### 3.5. Patients’ Perspectives on Factors Supporting Long-Term Recovery

Qualitative analysis of open-ended responses provided additional insight into patients’ perceptions of factors facilitating sustained recovery following adolescent anorexia nervosa. The principal themes identified are summarized in Table 7.

The most frequently reported theme concerned internal motivation and personal commitment to recovery, emphasizing the importance of accepting the need for treatment, maintaining perseverance, and taking responsibility for the recovery process. Participants repeatedly highlighted that successful recovery required an active personal decision to engage in treatment rather than passive participation.

Another prominent theme involved developing a sense of purpose and identity beyond the eating disorder. Respondents stressed the importance of rebuilding self-esteem, pursuing meaningful life goals, and redefining personal identity independently of body weight or appearance.

Trust in healthcare professionals and continued engagement with treatment were also identified as key facilitators of long-term recovery. Many participants emphasized the value of maintaining therapeutic relationships after discharge and following professional recommendations throughout the recovery process.

Additional themes included acceptance of support from family and friends, patience with the gradual nature of recovery, and recognition that recovery trajectories differ substantially between individuals. Several respondents emphasized that long-term improvement should be viewed as a continuous process rather than a single therapeutic outcome.

Collectively, these qualitative findings complement the quantitative analyses by demonstrating that successful long-term recovery extends beyond symptom remission and nutritional rehabilitation, encompassing psychological resilience, social support, and sustained personal engagement in the recovery process.

## 4. Discussion

### 4.1. Principal Findings

The present findings reinforce the growing recognition that long-term recovery following adolescent anorexia nervosa (AN) is a multidimensional process that cannot be adequately captured by anthropometric indicators alone. This conclusion is supported by the integration of objective clinical measures with psychological, behavioural, and patient-reported outcomes, including psychiatric comorbidity, orthorexic tendencies, caregiver perspectives, subjective health perception, and qualitative patient narratives. Although restoration of body weight remains essential for reversing the medical consequences of starvation, the present findings indicate that physiological stabilization alone should not be considered synonymous with complete recovery. Instead, long-term recovery should be viewed as a dynamic process encompassing sustained remission of eating-disorder symptoms, normalization of eating-related cognitions and behaviours, restoration of psychosocial functioning, improved quality of life, and recovery of personal autonomy [6,8,9]. The multidimensional recovery profile observed in the present cohort further demonstrates that clinically relevant aspects of recovery remain undetected when outcome assessment relies solely on anthropometric or diagnostic indicators. This interpretation is consistent with contemporary recommendations advocating comprehensive follow-up that integrates medical, nutritional, psychological, behavioural, functional, and patient-reported outcomes to provide a more accurate evaluation of long-term recovery [3,20,21].

One of the most clinically important findings was the clear dissociation between physical restoration and sustained psychological recovery. Although nutritional rehabilitation was substantial, with the mean BMI increasing from 13.4 kg/m^2^ during the acute phase of illness to 18.8 kg/m^2^ at long-term follow-up, approximately 60% of former patients continued to screen positive on the SCOFF questionnaire, suggesting that persistent eating-disorder symptoms coexisted with psychiatric comorbidity, orthorexic tendencies, and impaired subjective health perception. These findings emphasize that normalization of nutritional status should not be interpreted as evidence of complete recovery, as physical and psychological improvement appear to follow partially independent trajectories. Similar discrepancies have consistently been reported in longitudinal studies demonstrating that restoration of body weight often precedes remission of maladaptive cognitions, emotional distress, and dysfunctional eating behaviours, leaving many individuals vulnerable to persistent symptoms and relapse [8,22,23]. Importantly, our findings extend these observations by showing that persistent eating-disorder symptoms identified by the SCOFF screening questionnaire coexisted with psychiatric comorbidity, orthorexic tendencies, and impaired subjective health perception, indicating that incomplete recovery affects multiple interrelated domains rather than persistent eating-disorder symptoms alone.

Beyond confirming previous observations, the present study extends current knowledge by demonstrating that incomplete psychological recovery encompasses multiple interrelated domains that are rarely evaluated simultaneously within a single long-term follow-up study. Whereas previous investigations primarily focused on diagnostic remission, body weight restoration, or relapse rates, our findings demonstrate that persistent eating-disorder symptoms frequently coexist with orthorexic tendencies, psychiatric comorbidity, impaired subjective health perception, and discrepancies between objective clinical improvement and patients’ own perceptions of recovery. Integrating quantitative assessments with qualitative patient narratives provided complementary insight into how objective clinical outcomes are experienced in everyday life, highlighting dimensions of recovery often overlooked in conventional follow-up studies. To our knowledge, relatively few long-term investigations have combined clinical, psychological, behavioural, and experiential outcomes within the same cohort, providing a more comprehensive characterization of recovery after adolescent anorexia nervosa [6,21].

The clinical implications of these findings extend beyond evaluating treatment outcomes and have direct relevance for the long-term management of individuals recovering from adolescent anorexia nervosa. The observed dissociation between nutritional rehabilitation and psychological recovery indicates that follow-up based predominantly on anthropometric indicators may fail to identify patients who continue to experience clinically significant psychological difficulties despite satisfactory physical recovery. Our findings therefore support a longitudinal, multidisciplinary model of care in which nutritional status is interpreted alongside eating-disorder psychopathology, psychiatric comorbidity, cognitive and behavioural functioning, quality of life, and patient-reported outcomes. Such an approach is consistent with contemporary recommendations advocating individualized follow-up strategies that acknowledge the heterogeneous nature of recovery trajectories and emphasize the need for continued psychological support after medical stabilization [3]. Comprehensive multidimensional assessment may facilitate earlier identification of individuals at increased risk of incomplete recovery or relapse, enabling interventions to be tailored according to residual psychological needs rather than body weight alone.

Overall, the present findings support a broader conceptualization of long-term recovery following adolescent anorexia nervosa, emphasizing that nutritional rehabilitation represents only one component of a complex and evolving recovery process. The coexistence of satisfactory nutritional recovery with persistent eating-disorder symptoms, psychiatric comorbidity, orthorexic tendencies, and heterogeneous patient-reported experiences demonstrates that recovery is multidimensional and that its biological, psychological, behavioural, and psychosocial components do not necessarily improve in parallel. These observations challenge traditional weight-centred models of outcome assessment and reinforce the need for comprehensive, patient-centred frameworks capable of capturing the complexity of sustained recovery. Such an approach not only provides a more meaningful evaluation of long-term treatment outcomes but also facilitates the identification of individuals who may benefit from continued psychological support despite satisfactory nutritional rehabilitation. This multidimensional perspective provides the conceptual foundation for the following sections addressing persistent eating-disorder symptoms and orthorexic tendencies.

### 4.2. Persistence of Eating-Disorder Symptoms Despite Nutritional Recovery

One of the most clinically important findings of the present study was the persistence of eating-disorder symptoms despite substantial nutritional rehabilitation during long-term follow-up. Although participants achieved marked improvement in nutritional status, approximately 60% continued to screen positive on the SCOFF questionnaire, suggesting that psychological recovery remained incomplete despite satisfactory physical restoration. Rather than representing an unexpected observation, this dissociation reinforces the growing recognition that nutritional rehabilitation and psychological recovery follow partially independent trajectories. Previous longitudinal studies have consistently demonstrated that restoration of body weight often precedes remission of maladaptive cognitions, emotional distress, and dysfunctional eating behaviours, leaving many individuals vulnerable to persistent symptoms and subsequent relapse [8,22,23]. Importantly, our findings extend these observations by showing that persistent eating-disorder symptoms identified by the SCOFF screening questionnaire coexisted with psychiatric comorbidity, orthorexic tendencies, and heterogeneous perceptions of health, suggesting that incomplete recovery affects multiple domains of functioning rather than eating-disorder symptoms alone.

The persistence of eating-disorder symptoms identified by the SCOFF screening questionnaire observed in the present cohort is unlikely to reflect delayed recovery alone and may instead indicate the continued influence of biological and psychological mechanisms that remain active beyond nutritional rehabilitation. One plausible explanation is that prolonged starvation induces adaptive changes within neural systems involved in reward processing, cognitive control, emotional regulation, and habit formation, many of which appear to be only partially reversible after weight restoration [24,25]. These neurobiological adaptations are closely intertwined with enduring psychological characteristics frequently associated with anorexia nervosa, including cognitive rigidity, perfectionism, intolerance of uncertainty, and compulsive behavioural patterns, which may continue to reinforce maladaptive eating-related cognitions despite satisfactory physical recovery [14,26,27]. The present findings are consistent with these models, as persistent eating-disorder symptoms were accompanied by orthorexic tendencies, psychiatric comorbidity, and impaired subjective health perception, suggesting that these domains may share common underlying mechanisms rather than represent independent clinical phenomena. Nevertheless, the present study was not designed to determine the relative contributions of neurobiological vulnerability, premorbid personality traits, treatment history, or environmental influences. Future longitudinal studies integrating clinical, neurocognitive, and biological assessments are therefore needed to clarify the mechanisms responsible for persistent psychological symptoms following nutritional recovery.

Our findings are consistent with previous evidence indicating that complete psychological remission is achieved considerably less frequently than somatic recovery. Although the reported prevalence of persistent symptoms varies across studies because of differences in diagnostic criteria, follow-up duration, and definitions of recovery, residual psychopathology has consistently been associated with poorer psychosocial functioning, reduced quality of life, and an increased risk of relapse [5,22,23]. Our findings extend these observations by demonstrating that persistent eating-disorder symptoms identified by the SCOFF screening questionnaire frequently coexisted with orthorexic tendencies, psychiatric comorbidity, impaired subjective health perception, and discrepancies between objective nutritional recovery and patients’ own evaluation of health. These findings suggest that persistent eating-disorder symptoms should be interpreted within the broader context of incomplete psychological recovery rather than as an isolated clinical outcome.

The clinical implications of persistent eating-disorder symptoms extend beyond relapse prevention and should inform the long-term organization of post-treatment care. Our findings indicate that a substantial proportion of individuals who achieved satisfactory nutritional rehabilitation continued to experience clinically significant psychological symptoms, suggesting that follow-up based primarily on anthropometric indicators may fail to identify patients requiring ongoing intervention. These observations support a longitudinal, multidisciplinary model of care in which routine assessment includes screening for persistent eating-disorder symptoms, psychiatric comorbidity, cognitive and behavioural functioning, quality of life, and patient-reported outcomes alongside nutritional status. Such an approach is consistent with contemporary recommendations emphasizing individualized follow-up and recognition of the heterogeneous trajectories of recovery after anorexia nervosa [3,20,21]. Comprehensive monitoring may facilitate earlier identification of individuals at increased risk of incomplete psychological recovery, allowing therapeutic interventions to be adapted according to residual clinical needs rather than body weight alone. These findings underscore the need for continued psychological follow-up after nutritional rehabilitation.

Collectively, the present findings demonstrate that persistent eating-disorder symptoms identified by the SCOFF screening questionnaire represent an important component of long-term recovery rather than merely a residual consequence of the acute phase of anorexia nervosa. The coexistence of psychological symptoms with satisfactory nutritional rehabilitation highlights the need for continued assessment beyond anthropometric recovery. Persistent eating-disorder symptoms also coexisted with psychiatric comorbidity, impaired subjective wellbeing, and maladaptive eating-related behaviours. This broader perspective provides the rationale for examining orthorexic tendencies in greater detail, as they may represent an additional expression of unresolved psychological vulnerability rather than evidence of successful adaptation following nutritional rehabilitation.

### 4.3. Orthorexic Tendencies as a Marker of Incomplete Psychological Recovery

One of the most distinctive findings of the present study was the strong association between orthorexic tendencies and persistent eating-disorder symptoms during long-term follow-up. Among all variables examined, orthorexic behaviours showed one of the strongest associations with positive SCOFF screening results, suggesting that excessive preoccupation with healthy eating may represent a marker of incomplete psychological recovery rather than an isolated eating-related phenomenon. These findings support the growing view that orthorexic behaviours should be interpreted within the clinical context in which they emerge, particularly among individuals with a history of anorexia nervosa, where rigid dietary rules may represent the continuation or transformation of previous eating-disorder psychopathology rather than a distinct clinical entity [28,29,30]. Unlike observations from non-clinical populations, our findings suggest that orthorexic tendencies following anorexia nervosa may have a distinct clinical significance. Consequently, orthorexic behaviours should be regarded as clinically meaningful indicators of incomplete recovery that warrant careful assessment during long-term follow-up [28,31].

The close association between orthorexic tendencies and persistent eating-disorder symptoms observed in the present cohort is unlikely to be explained solely by an increased interest in healthy eating. Instead, these behaviours may reflect the persistence of cognitive and behavioural processes established during the acute phase of anorexia nervosa that continue to influence eating-related decision-making despite nutritional rehabilitation. One plausible explanation is that enduring traits such as cognitive rigidity, perfectionism, intolerance of uncertainty, and compulsive self-control become redirected toward socially acceptable dietary practices rather than disappearing after weight restoration [14,27,32]. Contemporary neurocognitive models further suggest that prolonged starvation promotes maladaptive habit formation and altered reward processing, increasing the likelihood that rigid eating behaviours persist even after medical stabilization [24,27,32]. Our findings are consistent with this interpretation, as orthorexic tendencies rarely occurred in isolation. Nevertheless, the present study cannot determine whether orthorexic behaviours represent a direct continuation of anorexia nervosa, a maladaptive coping strategy, or a distinct post-treatment phenotype, highlighting the need for prospective longitudinal studies integrating psychological and neurobiological assessment.

The interpretation of orthorexic tendencies in individuals with a history of anorexia nervosa remains a matter of ongoing debate, and the present findings provide additional evidence to inform this discussion. Some investigators have suggested that an increased focus on healthy eating following recovery may represent an adaptive strategy supporting long-term health and relapse prevention rather than persistent psychopathology [33,34]. An alternative perspective proposes that rigid adherence to dietary rules reflects the transformation rather than the resolution of anorexia nervosa, with concerns about body weight gradually replaced by excessive preoccupation with food purity and nutritional quality [15,28]. Our findings appear to support the latter interpretation. However, these findings should be interpreted with caution because orthorexic tendencies were assessed using study-specific questions rather than a validated orthorexia questionnaire. This decision was made intentionally to reduce respondent burden and maximize participation after the prolonged follow-up period, as the primary aim of the study was to evaluate long-term clinical outcomes rather than to diagnose orthorexia nervosa. Consequently, the findings related to orthorexic tendencies should be interpreted as exploratory and require confirmation in future studies using validated assessment instruments. Orthorexic tendencies were not observed as isolated health-oriented behaviours but occurred alongside persistent eating-disorder symptoms, psychiatric comorbidity, and poorer subjective health perception, suggesting that they are more likely to represent manifestations of unresolved psychological vulnerability than indicators of successful recovery. Nevertheless, these conclusions should be interpreted cautiously because the absence of universally accepted diagnostic criteria for orthorexia nervosa continues to limit comparisons across studies and complicates the distinction between pathological and non-pathological health-oriented eating behaviours [28,29,30].

The clinical implications of these findings extend beyond recognizing orthorexic behaviours as a potential comorbidity and suggest that they should be routinely considered during long-term follow-up of individuals recovering from anorexia nervosa. The observed association between orthorexic tendencies and persistent eating-disorder symptoms suggests that rigid health-oriented eating behaviours may signal incomplete psychological recovery rather than successful adaptation to a healthy lifestyle. Clinicians should therefore evaluate orthorexic behaviours within the broader psychological and clinical context instead of interpreting them solely as evidence of nutritional awareness or dietary discipline. Incorporating structured assessment of orthorexic symptoms into multidisciplinary follow-up may facilitate earlier identification of patients at increased risk of persistent psychopathology and allow timely adjustment of psychological interventions before clinically significant deterioration occurs.

Collectively, the present findings suggest that orthorexic tendencies should not be interpreted as an isolated behavioural phenomenon but rather as one manifestation of incomplete psychological recovery following anorexia nervosa. Their close association with persistent eating-disorder psychopathology, psychiatric comorbidity, and poorer subjective health perception indicates that rigid health-oriented eating behaviours may reflect continuing psychological vulnerability despite satisfactory nutritional rehabilitation. These observations reinforce the importance of evaluating orthorexic symptoms as part of long-term psychological follow-up. Importantly, understanding the clinical significance of orthorexic behaviours also requires consideration of patients’ own perceptions of recovery, as objective measures alone cannot fully capture the complexity of post-treatment adaptation. This perspective provides the rationale for the following section, which explores patients’ experiences of recovery and the therapeutic factors they identified as most meaningful during long-term follow-up.

### 4.4. Patients’ Perspectives on Recovery and Therapeutic Factors

An important contribution of the present study is the incorporation of patients’ own perspectives into the evaluation of long-term recovery following adolescent anorexia nervosa. While the quantitative findings demonstrated persistent psychological vulnerability despite satisfactory nutritional rehabilitation, the qualitative narratives provided valuable insight into how former patients understood and experienced recovery. Participants consistently described recovery as a gradual process involving the restoration of autonomy, emotional stability, interpersonal relationships, self-acceptance, and engagement in everyday life rather than simply achieving a target body weight or the absence of eating-disorder symptoms. These observations reinforce the growing recognition that patient-defined recovery extends beyond traditional clinical outcomes and encompasses dimensions rarely captured by anthropometric or symptom-based measures alone [11,21,35].

The qualitative narratives further demonstrated that patients frequently evaluated recovery using criteria that differed substantially from conventional clinical outcome measures. Rather than focusing primarily on body weight or symptom remission, participants emphasized regaining a sense of identity, autonomy, emotional balance, meaningful relationships, and the ability to engage fully in everyday life. These observations suggest that recovery is experienced not as a discrete clinical endpoint but as an evolving process of personal and psychosocial reconstruction. This interpretation is consistent with recovery-oriented models of care, which emphasize that sustainable recovery extends beyond symptom reduction and incorporates the individual’s own goals, values, and lived experiences [11,21,36]. The convergence between participants’ narratives and the quantitative findings further supports the view that psychological adaptation and quality of life may improve independently of anthropometric recovery. These findings therefore underscore the value of integrating qualitative assessment into long-term follow-up, particularly when evaluating outcomes that cannot be adequately captured by standardized clinical measures.

The qualitative findings also highlight the importance of incorporating patients’ perspectives into the interpretation of long-term treatment outcomes rather than considering them merely as complementary observations. Participants frequently identified feeling understood, rebuilding trust, regaining independence, and developing a stable sense of self as pivotal milestones in recovery, despite these dimensions remaining largely invisible within conventional clinical assessments. These findings suggest that patient narratives provide unique insight into the subjective meaning of recovery and help explain why objective indicators of improvement do not always correspond to patients’ own perceptions of health and wellbeing. This interpretation is consistent with person-centred models of care, which emphasize shared decision-making, therapeutic relationships, and recognition of individual recovery goals as integral components of effective treatment [1,11,36]. Rather than replacing standardized outcome measures, qualitative assessment enriches their interpretation by identifying dimensions of recovery that are difficult to quantify but highly relevant to long-term functioning and quality of life.

The present findings also illustrate the limitations of evaluating treatment success exclusively through standardized clinical outcomes. Although body weight restoration, symptom reduction, and diagnostic remission remain fundamental indicators of treatment effectiveness, the present data demonstrate that these measures do not necessarily reflect how recovery is experienced by former patients. Participants consistently attributed equal or greater importance to improvements in autonomy, emotional resilience, interpersonal functioning, and overall quality of life than to changes in body weight or eating-disorder symptoms. These observations support the growing emphasis on patient-reported outcomes (PROMs) and person-centred outcome assessment, which advocate integrating patients’ experiences with conventional clinical indicators to achieve a more comprehensive evaluation of long-term recovery [21,37,38]. Rather than replacing objective assessment, patient-reported outcomes complement traditional measures by capturing dimensions of recovery that are directly relevant to everyday functioning, wellbeing, and sustained adaptation following treatment. Consequently, incorporating qualitative assessment together with validated patient-reported outcome measures into long-term follow-up may improve both clinical decision-making and the evaluation of treatment effectiveness.

Collectively, the qualitative findings reinforce the view that long-term recovery from anorexia nervosa cannot be fully understood without integrating patients’ perspectives with conventional clinical assessment. While objective indicators remain essential, the present findings support a person-centred approach in which patient-reported experiences complement clinical assessment to provide a more comprehensive understanding of long-term recovery. These observations support a person-centred approach to follow-up in which patient-reported experiences complement objective clinical evaluation, facilitating a more comprehensive understanding of long-term recovery. This integrated perspective provides the foundation for the broader clinical implications, strengths, limitations, and future directions discussed in the following section.

### 4.5. Strengths, Limitations and Future Directions

The present study has several methodological strengths that enhance both the robustness and clinical relevance of its findings. Most importantly, the long-term follow-up of individuals treated for adolescent anorexia nervosa enabled evaluation of recovery well beyond the acute phase of illness, capturing outcomes that are rarely addressed in short-term investigations. The mixed-methods design further strengthened the study by integrating quantitative clinical assessment with qualitative patient narratives, thereby providing complementary perspectives on long-term recovery. In addition, the simultaneous evaluation of nutritional status, eating-disorder psychopathology, orthorexic tendencies, psychiatric comorbidity, subjective health perception, caregiver perspectives, and qualitative experiences allowed recovery to be examined as a multidimensional process rather than a single clinical endpoint. This comprehensive approach reflects the growing recognition that meaningful long-term outcomes extend beyond anthropometric or diagnostic measures alone and provides a broader understanding of recovery following adolescent anorexia nervosa [11,21,39]. Several limitations should be considered when interpreting these findings. First, the retrospective study design and relatively modest sample size may limit the generalizability of the results, although substantial attrition is an inherent challenge in long-term follow-up studies involving individuals treated during adolescence. Second, psychological outcomes were assessed primarily using self-reported questionnaires and retrospective qualitative interviews, both of which may be influenced by recall bias and social desirability. Nevertheless, the consistency between quantitative findings and qualitative narratives strengthens the credibility of the observed patterns and reduces the likelihood that the principal conclusions reflect methodological artefacts alone. Furthermore, although the study adopted a multidimensional approach to recovery assessment, biological markers, neurocognitive measures, and repeated longitudinal assessments were not available, limiting insight into the mechanisms underlying persistent psychological vulnerability. In addition, orthorexic tendencies were assessed using study-specific questions rather than a validated orthorexia questionnaire. This decision was made intentionally to reduce respondent burden and maximize participation after the prolonged follow-up period, as the primary aim of the study was to evaluate long-term clinical outcomes rather than to diagnose orthorexia nervosa. Consequently, findings related to orthorexic tendencies should be interpreted as exploratory and require confirmation using validated assessment instruments in future studies.

Finally, because the cohort originated from a single specialized treatment centre, caution is warranted when generalizing these findings to other healthcare systems or populations. Despite these limitations, the mixed-methods design provides a comprehensive and clinically meaningful evaluation of long-term recovery that is rarely achieved in follow-up studies of anorexia nervosa.

The present findings have important implications for both clinical practice and future research. They support a broader conceptualization of recovery following adolescent anorexia nervosa, emphasizing that successful long-term outcomes cannot be adequately defined by nutritional rehabilitation alone. The integration of anthropometric, psychological, behavioural, functional, and patient-reported outcomes demonstrated that persistent eating-disorder symptoms, orthorexic tendencies, psychiatric comorbidity, and patients’ own perceptions of recovery represent complementary dimensions of long-term recovery. These observations reinforce the importance of multidisciplinary, recovery-oriented models of care that recognize the heterogeneous trajectories of recovery and prioritize sustained psychological adaptation alongside physical restoration.

Future longitudinal studies should integrate clinical, neurocognitive, biological, and patient-reported outcome measures to better understand the mechanisms underlying incomplete recovery and to improve individualized prediction of long-term outcomes. Particular attention should be given to the role of orthorexic tendencies, as their strong association with positive SCOFF screening results observed in the present study suggests that they may represent a clinically meaningful indicator of unresolved psychological vulnerability.

Ultimately, adopting a multidimensional approach to recovery assessment may facilitate more personalized treatment strategies, improve continuity of care following discharge, and enhance the evaluation of long-term therapeutic outcomes in individuals recovering from anorexia nervosa.

## 5. Conclusions

Long-term recovery following adolescent anorexia nervosa cannot be adequately defined by nutritional rehabilitation alone. Although substantial improvement in nutritional status was observed during follow-up, a considerable proportion of participants continued to exhibit persistent eating-disorder psychopathology, highlighting the dissociation between physical and psychological recovery. Orthorexic tendencies demonstrated a particularly strong association with ongoing eating-disorder symptomatology, suggesting that an excessive preoccupation with healthy eating may represent a clinically relevant marker of incomplete psychological recovery rather than successful remission.

The integration of quantitative findings with patient- and caregiver-reported experiences further demonstrated that recovery extends beyond symptom reduction and encompasses psychological wellbeing, social functioning, and subjective perceptions of health. These findings support contemporary multidimensional models of recovery that incorporate physical, psychological, behavioural, functional, and patient-reported outcomes into long-term follow-up and treatment evaluation.

Overall, the present study highlights the importance of adopting comprehensive, patient-centred approaches to the assessment of long-term outcomes following adolescent anorexia nervosa. Future longitudinal studies involving larger and more diverse cohorts are warranted to validate these findings and to identify factors that promote sustained psychological recovery alongside nutritional rehabilitation.

## Figures and Tables

**Table 1:** Clinical Characteristics and Long-Term Outcomes of the Study Cohort.

Clinical Parameter	Respondents (N = 40)
Mean age at symptom onset (years)	14.5
Mean time from symptom onset to first hospitalization (years)	1.2 (~14 months)
Lowest recorded BMI, kg/m^2^	13.4
Current BMI at follow-up, kg/m^2^	18.8

**Table 2:** Clinical and Behavioral Factors Associated with Persistent Eating-Disorder Psychopathology During Long-Term Follow-Up.

Prognostic Variable	Statistical Result	Interpretation
Preoccupation with healthy eating (orthorexic behaviors) and positive SCOFF screening Presence of comorbid disorders (depression) and positive SCOFF screening Positive self-rated health and negative SCOFF screening	*p* < 0.001; Cramer’s V > 0.800	Very strong unfavorable prognostic factor (orthorexic pattern)
	Cramer’s V = 0.690	Strong unfavorable prognostic factor (psychiatric comorbidity)
	*p* = 0.006; Cramer’s V = 0.626	Strong protective factor

Note. *p* values were calculated using Fisher’s exact test. The strength of associations was assessed using Cramér’s V and interpreted according to the criteria described in the Section 2.5.

**Table 3:** Association Between Orthorexic Tendencies and Positive SCOFF Screening Results.

Preoccupation with Healthy Eating	Negative SCOFF *n* (%)	Positive SCOFF *n* (%)	Total
No	8 (80.0)	2 (20.0)	10
Yes	0 (0.0)	12 (100.0)	12
Total	8	14	22

Note. *p* values were calculated using Fisher’s exact test. The strength of associations was assessed using Cramér’s V and interpreted according to the criteria described in the Section 2.5.

**Table 4:** Association Between Psychiatric Comorbidity and Orthorexic Tendencies.

Comorbid Disorders	No Preoccupation *n* (%)	Preoccupation Present *n* (%)	Total
No	9 (75.0)	3 (25.0)	12
Yes	1 (10.0)	9 (90.0)	10
Total	10	12	22

Note. *p* values were calculated using Fisher’s exact test. The strength of associations was assessed using Cramér’s V and interpreted according to the criteria described in the Section 2.5.

**Table 5:** Association Between Self-Rated Health and Positive SCOFF Screening Results.

Self-Rated Health	Negative SCOFF *n* (%)	Positive SCOFF *n* (%)	Total
No	0 (0.0)	9 (100.0)	9
Yes	8 (61.5)	5 (38.5)	13
Total	8	14	22

Note. *p* values were calculated using Fisher’s exact test. The strength of associations was assessed using Cramér’s V and interpreted according to the criteria described in the Section 2.5.

**Table 6:** Integrated Evaluation of the Perceived Effectiveness of Inpatient Therapeutic Interventions.

Therapeutic Intervention	Mean Rating (1–5)	Effectiveness Category
Pharmacotherapy	3.97	Highest perceived effectiveness
Group psychotherapy	3.96	Highest perceived effectiveness
Regular body weight monitoring	3.91	High effectiveness
Therapeutic community	3.75	Moderate effectiveness
Therapeutic contract	3.75	Moderate effectiveness
Individual psychotherapy	3.64	Moderate effectiveness
Diet prescribed by healthcare professionals	3.60	Moderate effectiveness
Meal supervision	3.51	Moderate effectiveness
Temporary separation from family	3.19	Lowest perceived effectiveness
Family therapy	3.16	Lowest perceived effectiveness

**Table 7:** Major Themes Identified in Former Patients’ Recommendations for Long-Term Recovery Following Adolescent Anorexia Nervosa.

Theme	Frequency of Mentions	Illustrative Quotations
Internal motivation and commitment to recovery	8–10	‘You either want recovery or you don’t.’ ‘Accept that you need help.’
Finding purpose and identity beyond the illness	5–6	‘Find meaning in life.’ ‘Beauty is not thinness.’
Trust in healthcare professionals	5–7	‘Follow professional advice.’ ‘Trust the treatment team.’
Allowing oneself to recover	4–5	‘Accept help.’ ‘Take small steps.’
Social support and relationships	4–5	‘Stay connected with others.’
Continuation of treatment after discharge	3–4	‘Continue psychotherapy.’
Recovery is an individual process	4–5	‘Every case is different.’
Persistence and patience	3–4	‘Do not give up.’
Other recommendations	2–3	‘Listen to yourself.’

## Data Availability

All data and materials are included in the manuscript.
